# Identification and molecular characterization of non-polio enteroviruses from children with acute flaccid paralysis in West Africa, 2013–2014

**DOI:** 10.1038/s41598-017-03835-1

**Published:** 2017-06-19

**Authors:** Maria D. Fernandez-Garcia, Ousmane Kebe, Aichatou D. Fall, Kader Ndiaye

**Affiliations:** Institut Pasteur, Dakar, Senegal

## Abstract

Besides polioviruses, non-polio enteroviruses (NPEVs) may also be associated with acute flaccid paralysis (AFP). Because poliomyelitis is on the verge of eradication, more attention should be paid to study NPEVs from non-polio AFP cases and their epidemic patterns. In West African countries the epidemiology of NPEVs remains largely unexplored. We investigated the genetic diversity, frequency, circulation patterns, and molecular epidemiology of NPEVs in seven West African countries by analyzing retrospectively a panel of 3195 stool samples from children with AFP collected through routine poliomyelitis surveillance activities between 2013 and 2014. VP1 sequencing and typing on 201 isolates revealed 39 NPEV types corresponding to EV-A (6.9%), EV-B (90.5%), EV-C (2%) and EV-D (0.5%) species. Echoviruses were isolated most frequently with 138 cases (68.6%), followed by coxsackievirus group B with 35 cases (17.4%). No single NPEV type was remarkably dominant. Interestingly, several rarely described types with limited detection worldwide were identified (EVA76, EVA119, EVB75, EVB77, EVB97, EVC99, CVA20, CVA21 and EVD94). This study demonstrates the extensive diversity and diverse circulation patterns of NPEVs from AFP surveillance and highlights the need to formulate effective long-term strategies to monitor NPEV circulations in West Africa.

## Introduction

Enteroviruses (EVs) (family *Picornaviridae*, genus *Enterovirus*), are common human pathogens that circulate globally affecting the population in an endemic or epidemic pattern^1^. EV infections are an important public health problem as they result in considerable morbidity and occasionally in mortality. Only in the United States it is estimated that they cause about 10 to 15 million infections and tens of thousands of hospitalizations each year^2^. EV infections are usually asymptomatic or only result in mild disease such as upper respiratory illness (common cold) or nonspecific febrile illness. However, in some cases, EVs are associated with severe and potentially fatal diseases that affect mostly infants and children such as aseptic meningitis (AM), myocarditis, encephalitis, or poliomyelitis-like acute flaccid paralysis (AFP)^1^.

EVs are small non-enveloped viruses with positive single-strand RNA genomes. The genome is approximately 7.5 kb and is composed of a single open reading frame (ORF) flanked by 5′ and 3′ untranslated regions (UTRs). The ORF encodes a single polyprotein that is cleaved by viral proteases to yield structural proteins VP1-VP4 and non-structural proteins (2A to 2C and 3A to 3D)^1^. Among the structural proteins, the VP1 is a capsid protein located mainly at the virion surface which bears major viral epitopes recognized by neutralizing antibodies. Current EV classification is based on the high nucleotide sequence divergence within the VP1 capsid-coding region, which has been shown to correspond with serotyping using neutralization tests^3^. EVs can be identified by comparison of the entire or partial VP1 sequence to a database of prototype strain sequences. According to the recommended criteria for the interpretation of VP1 sequence data, EVs are classified into the same type if they have more than 75% nucleotide (nt) similarity and 88% amino acid (aa) identity and into different types if they have less than 70% nt identity in this region^3, 4^.

Based on genomic characteristics, EVs infecting humans are classified into four species EV-A to EV-D including more than 110 types^5^. Poliovirus (PV) is the best-studied EV. Its infection is known to be associated with AFP, a clinical manifestation characterized by rapid onset of weakness and reduced muscle tone of an individual’s extremities^6^. AFP surveillance is therefore a key strategy for polio eradication as it is a sensitive instrument for detecting potential poliomyelitis cases and PV infections^6^. Through the years, polio cases have been reduced worldwide and eradicated in some parts of the world, leaving non-polio enteroviruses (NPEVs) as one of the associated causes of AFP. In countries without specific EV surveillance systems, AFP surveillance is therefore the only data source to gain a better understanding of NPEV circulations. Despite the vast distribution of NPEVs reported globally, a complete view of the epidemiology of these viruses in West Africa is yet to be established. Information on the occurrence of different NPEV types will be important to determine the extent to which these viruses are circulating and whether there are emerging, as yet unidentified, types circulating within the West African population.

To investigate the genetic diversity, frequency and molecular epidemiology of NPEVs in West Africa, we retrospectively analyzed NPEV cell culture isolates derived from stool specimens of AFP cases obtained through routine poliomyelitis surveillance activities at the World Health Organization’s Regional Polio Laboratory in Senegal during 2013–2014.

## Results

### NPEV isolation from AFP cases and molecular typing of isolates

Overall, 3195 stool samples were collected during a 2-year period from 1600 children with AFP and tested for NPEV isolation (table 1). Three hundred and ninety six samples (396/3195, 12.4%) were positive for NPEVs in RD cell culture and remained negative in L20B cells. Of the 396 NPEV-positive cases, 236 were NPEV-positive individual cases and 160 NPEV-positive second specimens. Only one NPEV isolate per patient was analyzed for typing. The VP1 typing method which is the most common method for molecular typing of EV was used to screen cell-culture supernatants^3, 7–9^. A total of 201 (201/236, 85.2%) could be successfully typed by RT-snPCR targeting part of the 3′-VP3 and the 5′-VP1 regions. Overall amplicons from 32 samples showed very low signal insufficient for sequencing reactions and 3 samples could not be amplified although they showed amplification in 5′UTR of the EV genome. In order to characterize the 201 NPEV isolates, the 5′ half of the VP1 genomic region (~400 nt) was selected for comparative nucleotide alignment with prototype strains available in GenBank. Based on the cutoff value for unambiguous typing (nt and aa identities between isolates and their prototype strains higher than 75% and 88.0%, respectively), the 201 isolates were classified into 39 types corresponding to EV-A, EV-B, EV-C and EV-D species (Table 2). Twenty-three NPEVs (23/201, 11.4%) belonging to eight EV types (E1, E6, E7, E11, E13, CVA20, CVA21 and EVC99) did not reach the cutoff values of 75% nt and/or 88% aa identities, displaying sequence similarities slightly lower to these values (Table 2). Because the 70–75% nt similarity in VP1 region is considered as a “grey zone” of molecular typing of EVs, we obtained the complete VP1 or P1 (capsid) region nt sequences to refine typing results. The full-length VP1 sequence of the 25 strains displayed sequence similarities higher than 75% and 88% with prototype strains at nt and aa levels (data not shown), confirming our previous typing results with the partial VP1 sequence.

**Table 1:** Frequency of AFP cases, NPEVs isolated, NPEVs typed, isolation rate and typing rate, West Africa, 2013–2014.

	AFP cases	N° stool samples received	N° NPEVs isolated	Total isolation rate (%)	N° isolates tested for typing	N° NPEVs typed	Total typing rate (%)
**Niger**	596	1192	161	13.5	95	79	83.1
**Senegal**	423	843	104	12.3	64	54	84.3
**Guinea-Conakry**	355	710	70	9.9	42	36	85.7
**Mauritania**	108	216	33	15.3	20	19	95
**Gambia**	64	127	14	11	7	7	100
**Guinea-Bissau**	49	97	14	14.4	8	6	75
**Cape Verde**	5	10	0	0	0	0	0
Total	**1600**	**3195**	**396**	**12.4**	**236**	**201**	**85.2**

**Table 2:** Frequency of different enterovirus types detected in stool specimens of AFP cases, 2013-2014, based on partial VP1 gene sequences. SEN Senegal; MAU Mauritania; GAM Gambia; GUB Guinea Bissau; GUI Guinea Conakry; NIG Niger.

Species	Type	N° of isolates													% of total	nt homologies (aa) between prototype strain and isolates (%)*	GenBank accession numbers
		SEN		MAU		GAM		GUB		GUI		NIG		Total			
		Isolation Year#															
		13	14	13	14	13	14	13	14	13	14	13	14	13 + 14			
A	Coxsackievirus A2									1				**1**	0.5	80.7 (93.6)	KY433790
	Coxsackievirus A4	1						1			1			**3**	1.5	84.3–86 (96.3)	KY433787; KY433789; KY433793
	Coxsackievirus A6										1			**1**	0.5	83.8 (94.7)	KY433795
	Coxsackievirus A14		1							1				**2**	1	82–83.5 (98.5)	KY433792; KY433794
	Coxsackievirus A16											1		**1**	0.5	75.3 (88.4)	KY433791
	Enterovirus A71		1		1					1		1		**4**	2	80.6–82.4 (93.6–94.9)	KT818793-96
	Enterovirus A76												1	**1**	1	84.6 (99.2)	KY433796
	Enterovirus A119											1		**1**	0.5	93.8 (97.3)	KY433788
Subtotal		3		1				1		5		4		**14**	6.9		
B	Coxsackievirus B2	1	2							1		1	1	**6**	3	84.6–86.4 (98.5–99.2)	KY433600-05
	Coxsackievirus B3												2	**2**	1	77.4–77.7 (96.1)	KY433718-19
	Coxsackievirus B4	1		3		1				2		2	1	**10**	5	80.6–82.3 (99.3)	KY433737-46
	Coxsackievirus B5	2						1		2	1	3	2	**11**	5.5	75.5–81.4 (93.3–95.5)	KY433747-57
	Coxsackievirus B6											4	2	**6**	3	77.3–79.1 (96.2–97)	KY433606-11
	Echovirus 1		6								2		1	**9**	4.5	70.8–81.1 (88.1–97)	KY433612-14; KY433697-702
	Echovirus 2								1		1			**2**	1	78.6–80.2 (93.9–94.7)	KY433728; KY433730
	Echovirus 3		1		1		1			2		3	1	**9**	4.5	78.6–82.7 (93.9–99.3)	KY433758-66
	Echovirus 6	3	1							1	1	7	2	**15**	7.5	74.4–77.6 (90.3–93.3)	KY433767-78; KY433703-05
	Echovirus 7	2			2	1				1		3	1	**10**	5	74.4–78.9 (90–91.6)	KY433615-22; KY433706-07
	Echovirus 11	3		4							2		3	**12**	6	76.7–82.9 (86.9–96.3)	KY433623-29; KY433708-12
	Echovirus 12		1							1		2	1	**5**	2.5	79.4–81.4 (97.7–99.2)	KY433630-34
	Echovirus 13		5							1		4	6	**16**	8	70.1–78.9 (84.1–92.4)	KY433635-45; KY433713-17
	Echovirus 14										1	1		**2**	1	79.5 (96.3–97.1)	KY433726; KY433733
	Echovirus 18				1								2	**3**	1.5	83.9–85.2 (97.7–98.5)	KY433734-36
	Echovirus 19		3				1				2		1	**7**	3.4	77.8–79.8 (84.6–89.7)	KY433646-52
	Echovirus 20		4	1				1			1	1	1	**9**	4.5	78.8–82.5 (93.3–97)	KY433653-61
	Echovirus 24	1	1	1		1		1		3			1	**9**	4.5	76.8–78.6 (96.2–97)	KY433662-70
	Echovirus 25		2			1			1				1	**5**	2.5	79.1–80.3 (93.3–94.8)	KY433720-24
	Echovirus 27									1				**1**	0.5	78 (88)	KY433729
	Echovirus 29		4		1					2		1		**8**	4.3	76–78.5 (91–94)	KY433779-86
	Echovirus 30	2	1		1						1		2	**7**	3.4	80.7–83.2 (94.8–97)	KY433671-77
	Echovirus 32				1							1	1	**3**	1.5	79.1–79.8 (93.3–94.8)	KY433727; KY433731-32
	Echovirus 33	1	1	1									3	**6**	3	77.6–79.1 (95.5–97)	KY433678-83
	Enterovirus B75					1					2	1	1	**5**	2.5	84.5–87.9 (97–98.5)	KY433684-88
	Enterovirus B77											1		**1**	0.5	80.6 (92.7)	KY433725
	Enterovirus B97		1		1								1	**3**	1.5	88.1–87.4 (96.9–99.2)	KY433689-91
Subtotal		49		18		7		5		31		72		**182**	90.5		
C	Coxsackievirus A20											1	1	**2**	1	76–79.8 (86.5–94)	KY433692-93
	Coxsackievirus A21		1											**1**	0.5	74.7 (99)	KY433695
	Enterovirus C99		1											**1**	0.5	81.7 (79.7)	KY433694
Subtotal		2										2		**4**	2		
D	Enterovirus D94												1	**1**	0.5	81.6 (94.5)	KY433696
Subtotal												1		**1**	0.5		
Total		54		19		7		6		36		79		**201**	100		

*Homology was calculated using the ~400 bp length of the 5′ end of VP1.

^#^Year code: 13 for 2013; 14 for 2014

### Analysis of diversity, frequency and periodicity of NPEVs

The 201 typed isolates belonged to EV-B (90.5%), EV-A (6.9%), EV-C (2%) and EV-D (0.5%) species. Echoviruses were isolated most frequently with 138 cases (68.6%), followed by coxsackievirus group B with 35 cases (17.4%). Among the 201 isolates, echovirus 6 (E6) and echovirus 13 (E13) were the most frequently detected (n = 16 and n = 15, respectively) followed by E11 (6%) and coxsackievirus B5 (CVB5) (5.5%). Although majority of E13 and E6 cases were reported from Niger, (E13 n = 10; E6 n = 9), no outbreaks were observed as cases were scattered throughout the study period and throughout the country. Strains belonging to 9 types (CVB4, E1, E3, E7, E19, E20, E24, E29, E30) each accounted for 3.4–5% of the characterized strains and 7 types (EVA71, CVB2, CVB6, E12, E25, E33, EVB75) represented 2–3%. The frequency of detection of each of the other types ranged from 0.5% to 1.5%.

**Figure 1:**
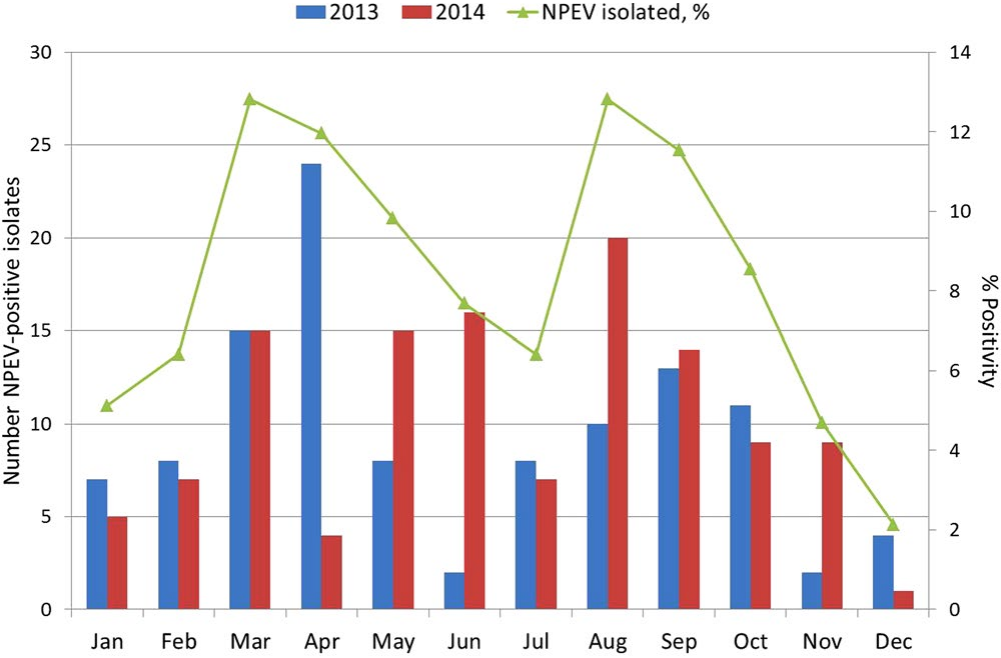
Monthly distribution of NPEV isolates from AFP cases, West Africa, 2013–2014.

The NPEV isolation rate during the study period ranged from 10.4% (192/1841) in 2013 to 15.9% (204/1354) in 2014 with a mean rate of 13.1%. Among AFP cases reported from Niger, Senegal, Guinea, Mauritania, Gambia, Guinea-Bissau and Cape Verde, NPEVs were detected in all countries but Cape Verde with isolation rates varying from 9.9% in Guinea to 15.3% in Mauritania (Table 1). The prevalence of EV-A, EV-B and EV-C infections was similar in 2013 and 2014 (8.3% vs 5.7%, 90.6% vs 89.6% and 1% vs 2.8%, respectively). Of the 39 different types identified, five (CVA2, CVA16, EVA119, CVB77 and E27) were only identified during 2013 (0.5% each) while CVA6, EVA76, CVB3, E1, E18, E19, EVB97, CVA21, EVC99 and EVD94 were only detected in 2014 (0.5–4.5%) (Table 2). CVB4, CVB5, E6, E7, E11, and E24 circulated more frequently during 2013 (7.3–11.5%), while E1, E13 and E19 were found more commonly in 2014 (6.6–10.4%). Strains belonging to EV-A and EV-B species were more frequently observed in Niger (28.5% and 39.3%, respectively), Senegal (21.4% and 26.7%, respectively) and Guinea (35.7% and 17.4%, respectively) probably because of the higher number of samples from these countries or higher circulation of those species (Table 2). Strains belonging to EV-C species were rarely observed, with one CVA21 and one EVC99 in Senegal and two CVA20 in Niger. Majority (3/4, 75%) were detected in samples from 2014. Only one strain from EV-D specie (EVD94) was found in Niger in 2014. The more geographically expanded types of NPEV strains included E24 (only type found in the six countries), CVB4, E3, E7 and E20 that were present in at least five countries. Types more frequently observed per country were E6 and E13 in Niger (n = 9 and n = 10 respectively), E1 and E13 in Senegal (n = 6 and n = 5, respectively), E11 and CVB4 in Mauritania (n = 4 and n = 3, respectively), E24 and CVB5 in Guinea (n = 3 for both) (Table 2). Although NPEV positive AFP cases were detected throughout the 2 years of study, seasonal variation was observed. NPEV infections showed a lower incidence from October to February (begin of dry cold season) (positivity rate range of 2.1–8.5%), while two incidence peaks were observed: one in March-April (end of dry cold season) (positivity rate of 12% and 12.8%) and other in August-September (rainy hot months) (positivity rate of 11.5% and 12.8%) (Fig. 1).

### Phylogenetic analysis

**Figure 2:**
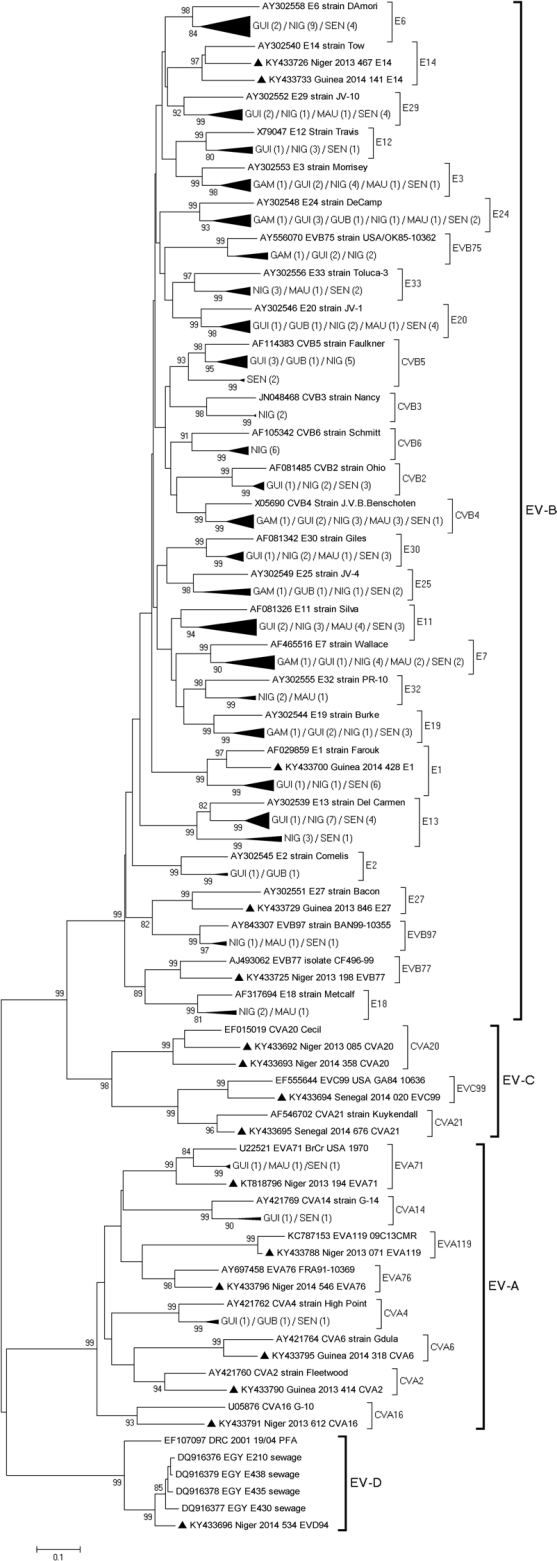
Phylogenetic analysis of 14 EV-A, 182 EV-B, 4 EV-C and 1 EV-D study strains and 43 international reference strains performed on ~400 bp-sized nucleotide sequence data set within the 5′-VP1 region. Phylogenetic trees were inferred with a Neighbor Joining algorithm that applied Kimura 2-parameter model after excluding gaps from the alignments. The reliability of the tree topologies was estimated by bootstrap analysis with 1000 replicates. Scale is shown at the bottom as substitutions per site. Evolutionary analyses were conducted with MEGA5 software. Study strains are indicated with black triangles. Study strains are indicated by the GenBank accession number, country, year of isolation and the internal laboratory code that corresponds to the serial number of the AFP case. Numbers in brackets indicate the number of study strains in the sub-cluster. SEN Senegal; MAU Mauritania; GAM Gambia; GUI Guinea Conakry; GUB Guinea Bissau; NIG Niger.

**Figure 3:**
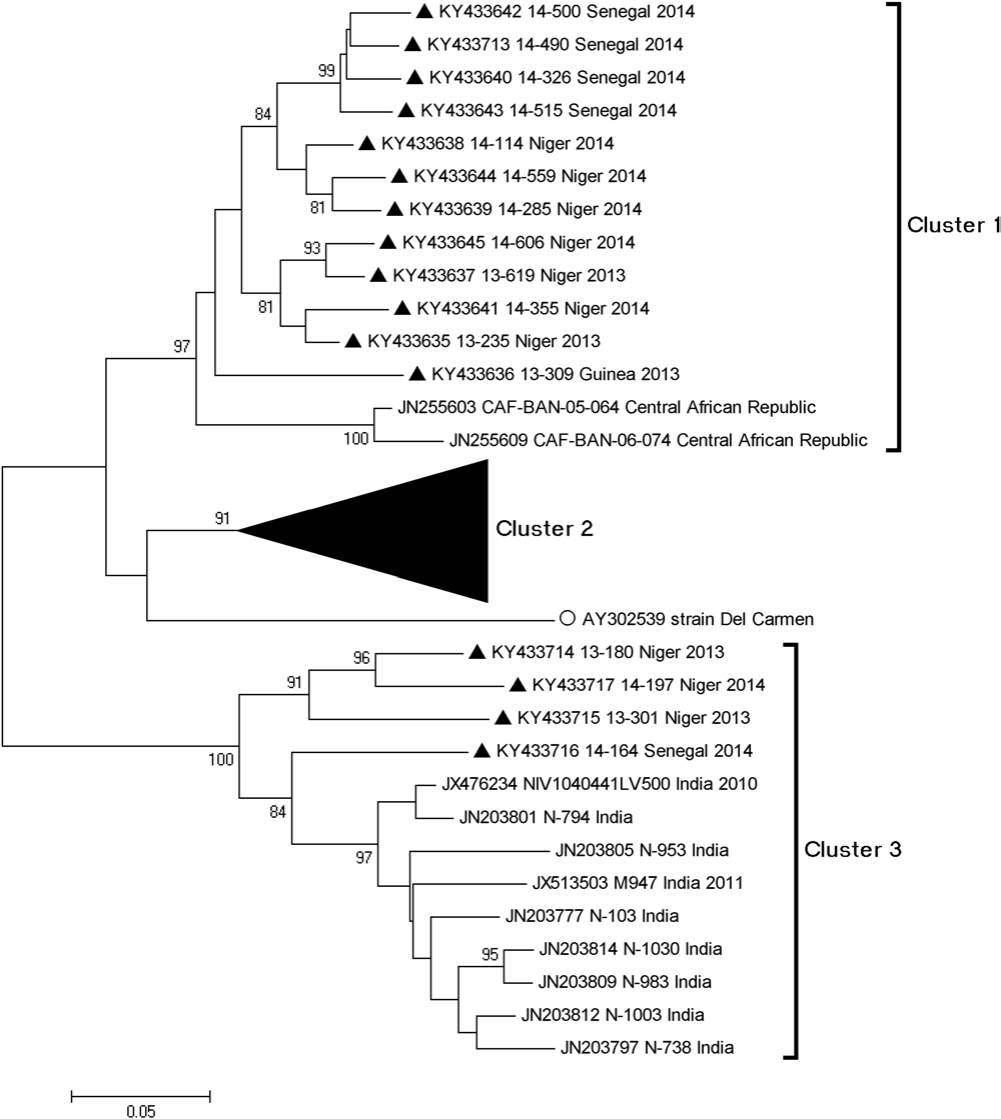
Phylogenetic analysis based on the VP1 nucleotide sequences (400 bp in length) of the 16 E13 study strains and a representative global set of 64 strains from GenBank database belonging to E13 type. Strains reported in this study are shown with black triangles. Cluster 2 (n = 52 strains) is compressed for clarity. Study strains are indicated by the GenBank accession number, laboratory code, country and year of isolation. Scale is shown at the bottom as substitutions per site.

**Figure 4:**
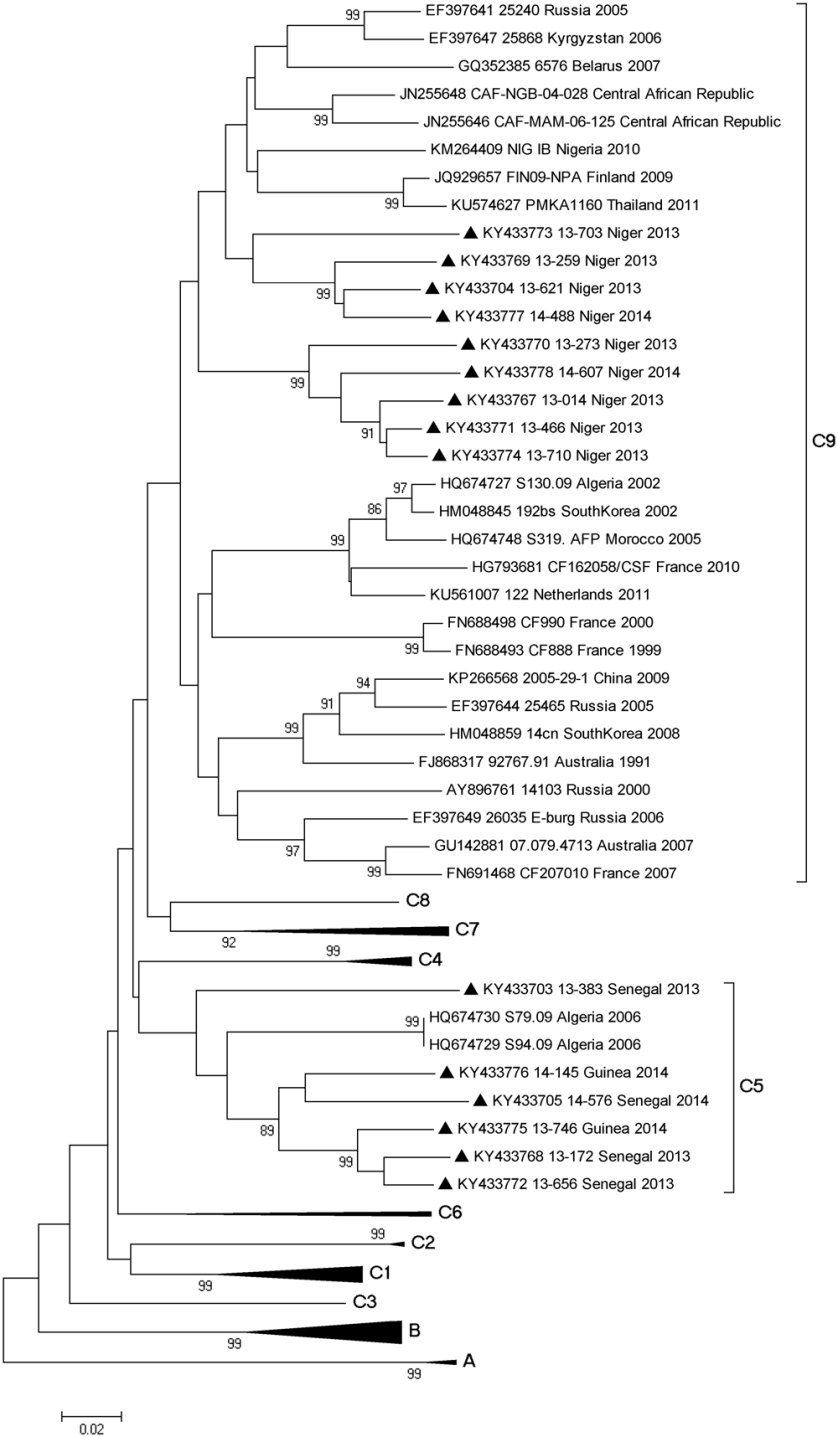
Phylogenetic analysis based on the VP1 nucleotide sequences (407 bp in length) of the 15 E6 study strains and a representative global set of 58 strains from GenBank database belonging to E6 type. Strains reported in this study are shown with black triangles. Study strains are indicated by the GenBank accession number, laboratory code, country and year of isolation. Scale is shown at the bottom as substitutions per site.

Classification of the strains into different types was also demonstrated by construction of phylogenetic trees using aligned partial or complete VP1 sequences. Study specimens of the same type clustered together with the respective prototype strains, supported by high bootstrap values, further confirming type identification (Fig. 2). Phylogenetic analysis of VP1 sequences of strains belonging to the most prevalent types E13 and E6 revealed that all isolates featured a wide range of genetic variability, expressed through segregation within different groups and sub-groups. For E13, the type more frequently detected, phylogenetic analysis indicated the existence of 3 different clusters that we designated 1–3 (Fig. 3). Twelve study strains from Senegal, Niger and Guinea fell in a bootstrap-supported cluster (cluster 1) with strains from Central African Republic (CAR) showing mean 89.1% (88.2–90%) nt and 97.5% (96.8–98.2%) aa similarities. The remaining appear to be closely related to E13 strains isolated from 2007 to 2011 in India (cluster 3) exhibiting mean 90.7% (89.9–91.5%) nt and 96.4% (95.6–97.6%) aa similarities. Low intratypic genetic similarity was observed between E13 study strains of both clusters (mean 73.8% (71.7–75.9%) nt and 86.7% (83.6–89.8%) aa homologies). Interestingly, when comparing all NPEV types to their respective homotypic prototype strains, E13 study isolates in cluster 3 were the more distantly related (mean 70.8% (70.1–71.5%) nt and 84.3% (84.1–84.5%) aa homologies) suggesting that these isolates may belong to a new group. However, submission of full P1 (capsid) region sequence to ICTV confirmed these isolates as E13 type (data not shown). Regarding E6, a notable observation was geographical segregation of West African strains within group C into the previously assigned sub-groups C5 and C9^10, 11^ (Fig. 4). In sub-group C5, strains isolated in Senegal and Guinea clustered together with Algerian strains isolated from AFP cases in 2006^12^ with a mean of 10.1% distance between each other and up to 24.4% divergence with the prototype E6 strain D’amori (group A). On the other hand, the nine E6 strains isolated in Niger clustered in sub-group C9 with strains from different countries and continents with a mean 10.8% divergence between each other and up to 23.6% divergence with the prototype (Fig. 4). Other prevalent types of EV-B species such as CVB4, CVB5, E1, E3, E7, E11, E20 and E24, and types of EV-A and EV-C species belonged to several lineages that have already been described for these types (data not shown). The unique EV-D isolate was typed as EVD94. Overall, six isolates of EVD94 have been reported so far, five from sewage in Egypt and one from an AFP case in Democratic Republic of Congo (DRC)^13, 14^. On the basis of the VP1 region, all isolates were closely related and formed a monophyletic group within the EV-D species (Fig. 2).

## Discussion

AFP surveillance is an essential strategy of the Global Polio Eradication Initiative. It aims to look for the circulation of wild-type and vaccine derived polioviruses in the community by systematic virus isolation from faeces samples of AFP cases^15^. In the context of PV surveillance, NPEVs are just considered a side product and do not require detailed testing for identification. However, in countries without specific NPEV surveillance systems, AFP surveillance provides the only source of information on their circulation. Retrospective investigation on NPEVs from AFP surveillance to understand their epidemiology has been widely carried out in different parts of the world, especially in India, China and Pakistan^16–21^. However, little is known about the epidemiology of NPEV infections in sub-Saharan Africa, in particular in West African countries. In this regard, the main goal of this two-year study was to investigate the frequency, circulation patterns, genetic diversity and molecular epidemiology of NPEVs from AFP surveillance in West African countries (Senegal, Mauritania, Gambia, Guinea Bissau, Guinea and Niger) between 2013 and 2014. For this aim, we first used susceptible cell culture to detect NPEV positivity. In our study, NPEVs accounted for about 15% (236/1600) of the total of AFP cases from 2013 to 2014 which is in agreement with the fact that the isolation rate of NPEV in most tropical countries typically exceeds 10%^22^. The analysis of the VP1 region has been widely carried out to type EV species that circulate in the population^9^. According to this strategy, our findings revealed a high diversity of NPEV strains that belonged to different species and types. Overall, 39 different NPEV types were identified. This observation was found to be close to that described in earlier studies of NPEV from AFP cases in CAR (n = 42), southern China (n = 45) and the Philippines (n = 47) by using similar methods^16, 23, 24^. In this work, types from the EV-A, EV-B, EV-C and EV-D species accounted for 6.9%, 90.5%, 2% and 0.5% of all isolates, respectively. This detection pattern EV-B > EV-A > EV-C > EV-D with high predominance of NPEV-B species is in line to that described previously in most NPEVs from AFP surveillance studies^17, 18, 21, 23, 25^. It is to be noted that the frequency of detected EV-C types (2%) is remarkably low compared to those in studies from Southern China, Philippines, Cameroon, Chad, Gabon and CAR (range 12.2–39.5%)^16, 23, 24, 26^. These differences may be attributed to the fact that the latter studies introduced the HEp-2 cell line which is known to be particularly suitable for the efficient isolation of CVA types of the EV-C species^26^. Our findings also reveal that the prevalence of EV-A, EV-B and EV-C infections was similar in 2013 and 2014 (8.3% vs 5.7%, 90.6% vs 89.6% and 1% vs 2.8%, respectively) suggesting a continuous pattern of circulation in West Africa. Echoviruses (n = 138) and Coxsackie-B viruses (n = 35) were the most frequent types in all countries of the study which is in accordance with other studies in Ghana, Nigeria, Pakistan, Eastern China, India and Philippines^17–19, 21, 24, 25, 27^ providing evidence of their high endemicity and worldwide distribution. Although a large number of types were detected, no single NPEV type was remarkably dominant. E13 and E6, which were the most frequently types detected in our study (n = 16 and n = 15, respectively), are among the most commonly detected EV worldwide and have been often reported in association with serious diseases in children such as AM and AFP^11, 28–35^. Between 2001 and 2002, E13 emerged as a predominant EV in the United States and in Japan causing numerous AM outbreaks^33^. Under AFP surveillance, E13 was also found the predominant NPEV in Northern India and China^16, 29^, and among the most prevalent types in DRC, CAR, South-Western India, and Philippines^13, 18, 23, 24^. E6 has been found to be prevalent in AFP cases from Nigeria, Eastern China, Pakistan, Philippines and Cameroon^17, 21, 24–26^. Compared to the genetically distant E13 isolates, the E6 isolates in West Africa were less divergent. All E6 isolates clustered within the previously reported group C^10, 11^. However, it is to be noted an apparent geographical segregation within African strains. Field strains from Niger (n = 9) cluster in sub-group C9 with strains isolated worldwide. However, study strains from Guinea and Senegal (n = 6) fell into sub-group C5 with strains isolated in Algeria suggesting a probable lineage circulating in the region. Similarly to E13 and E6, other prevalent echoviruses (E1, E3, E7, E11, E20, E24) were quite divergent (data not shown) suggesting that there are several transmission chains and that have been circulating with high endemicity for many years in West Africa.

The leading pathogens of hand, foot and mouth disease (HFMD) – EVA71 and CVA16 - are present in this work. CVA16 and EVA71 related to HFMD have been endemic in the Asia-Pacific region for decades causing outbreaks with high morbidity and mortality. Compared to other EVs, EV71 is more often associated with severe and sometimes fatal neurologic complications such as AFP. In recent years, CVA6 has also become one of the principal pathogens responsible for causing HFMD outbreaks in Europe, North America and Asia^36^. This study reports the detection of four EVA71 strains which are described further in Fernandez-Garcia *et al*.^37^, as well as one CVA6 and one CVA16. Among the four EVA71 strains, one belonged to the recently discovered genogroup E in Africa and the other three to genogroup C^37^. Sporadic reports demonstrated that other EV-A types such as CVA2, CVA4 and CVA14, all present in this study, can also be attributed to HFMD^38, 39^. Interestingly, up to our knowledge, in Africa, CVA2, CVA4, CVA6, CVA14 and CVA16 types were previously reported circulating in healthy children^25, 40–42^ but not in AFP cases. Because in China, several EV-A viruses were observed during AFP surveillance before the onset of large-scale HFMD outbreaks in 2007 and in 2008^17^ and given that the rapid spread of HFMD outbreaks in Asia appears to be associated with the emergence of new groups and sub-groups that result from the co-circulation and recombination events between EV-A types^43^, surveillance systems to monitor HFMD and detect potential novel recombinants should be introduced in Africa to prevent outbreaks.

A remarkable finding is the detection in this study of rare types belonging to all EV species A-D with limited detection worldwide (EVA76, EVA119, EVB75, EVB77, EVB97, EVC99, CVA20, CVA21 and EVD94). All but EVC99 have been sporadically isolated in AFP cases in the Central African subregion^13, 23, 26^. In Africa, EVC99 was previously detected in healthy children from North Cameroon and Nigeria and in a chimpanzee with AFP in DRC^26, 42, 44^. Similar to EVC99, EVA76 and EVA119 viruses have been shown to infect both humans and chimpanzees in Cameroon^45, 46^. Because countries where these viruses were reported (Niger for EVA76 and EVA119 and Senegal for EVC99) are home for nonhuman primates (NHP) and given the high exposure levels between humans and NHP in West and Central Africa^47, 48^, our finding support previous studies which argue interspecies transmission of these EVs and rise concern about primates as potential source of emerging EV infections^44–46^. Notably, EVD94 was the only species-D type detected in this study. The study strain EVD94 from Niger cluster with an isolate from DRC associated with AFP and sewage isolates from Egypt^14^, corroborating the fact that individuals shed virus into faeces, and hence into sewage which in turn serve as a source of infection to the population.

Some limitations which may bias the diversity data in our study should be considered. First, this is a 2-year study and thus, it might be difficult to identify over this limited time period those types that have constant circulation and those that are occasional and may have been imported. Second, the use of only one susceptible cell line during the NPEV isolation process may have led to selective growth of some types and not others since some CVA from EV-C species and some CVB are known to grow poorly on RD cells^49^. Of notice, although this study describes EV typing results from AFP surveillance, detection of a NPEV type during the course of AFP in stool samples does not confirm it as a causative agent of AFP as NPEVs are excreted in feces for a long period.

In conclusion, the present study reveals an extensive genetic diversity in NPEVs circulating in AFP cases in West Africa. Many of the NPEV types identified in this study (E13, E6, E11, E7, EVA71, CVA16, CVA6) are well known among the types described in AM and HFMD outbreaks commonly reported worldwide. Now that poliomyelitis is on the verge of eradication, data generated from this study argues for a better understanding of the role of NPEVs as non-polio AFP etiologies and highlights the need to formulate effective long-term strategies to monitor NPEV infections in Africa.

## Methods

### Ethics statement

This study is a component of the Global Polio Eradication Initiative. This study did not involve human participants, but the use of cell culture isolates of viruses recovered from stool specimens of AFP cases collected through routine poliomyelitis surveillance activities at the instigation of Word Health Organization (WHO) for public health purposes. All technical and ethical aspects were approved by WHO and Ministries of Health of countries concerned. The protocol and oral consent were determined as routine surveillance activity by the steering committee of WHO in compliance with all applicable National regulations governing the protection of human subjects. The methods were carried out in accordance with the principles of the Declaration of Helsinki.

### Specimens and virus isolation

Stool samples (n = 3195) were collected from 1600 patients aged <15 years and presenting with AFP from January 2013 to December 2014 through routine poliomyelitis surveillance activities in accordance with ethical guidelines of the programme. Samples coming from Niger, Senegal, Guinea, Mauritania, Gambia, Guinea-Bissau and Cape Verde were sent to the WHO-accredited Regional Reference Polio Laboratory in Senegal for processing according to the standard procedures of the WHO^50^. All cases in 2014 had two stool specimens while in 2013 it was majority (919/923, 99.6%) of cases. Specimens were collected 24–48 hours apart and within 14 days of onset of paralysis. Chloroform-extracted and clarified stool suspensions were inoculated in human rhabdosarcoma (RD) and human poliovirus receptor-CD155 expressing recombinant murine (L20B) cell lines. For samples showing no cytopathic effect (CPE) after 5 days, a blind passage in the same cell line was done using the supernatant of the inoculated negative cultures and microscopically checked for the next 5 days. Viral cultures positive in RD cells but negative after 10 days in L20B cells were re-passaged in L20B cells and examined for 5 days to exclude the possibility that they were polioviruses. Only viral cultures producing CPE in RD cells and not in L20B cells were considered to contain NPEVs. When CPE was obtained, infected cells were harvested and kept frozen (−20 °C) until typing. When two isolates originated from the two stool specimens of the same patient, only one isolate was analyzed for typing. Work with infectious viruses was carried out in a BSL-2 facility.

### RNA extraction and amplification of viral genome

Viral RNA was extracted from 140 µl of virus-positive cell culture supernatant using the QIAamp viral RNA mini kit (Qiagen, Germany) according to the manufacturer’s recommendations. For viral detection, RNA was reverse transcribed into cDNA and tested using a semi nested reverse-transcription-polymerase chain reaction (RT-snPCR) as previously described^50^. The first round of PCR amplification was carried out in a 20 µl reaction volume containing 5 µl of the cDNA product, 4 µl of the PCR buffer (Promega), 5 U of GoTaq DNA polymerase (Promega), 10 pmol of sense primer AMTH and 10 pmol of antisense primer GDCL^7^. This reaction mixture was amplified using the following conditions: 3 min at 94 °C followed by 40 cycles of 30 s at 94 °C, 30 s at 45 °C and 2 min at 60 °C, plus a final extension step at 60 °C for 10 min. For snPCR the reaction was achieved on 1 µl of the first amplification using primers 222/224 as previously described^9^. The complete VP1 sequences of 25 isolates were obtained by using virus-specific primers available upon request. In order to avoid cross-contamination, two negative controls were included in the RT-PCR: RNA extracted from normal RD cells and all the components of the reaction except for the template. Products were analyzed on BET-stained agarose gels and visualized under ultraviolet light. A positive RT-PCR reaction was expected to produce a 756-bp band.

### Genome sequencing and phylogenetic analysis

Amplified products were directly sequenced at Genewiz (Essex, United Kigdom) by the Sanger method using the same forward and reverse primers (224 and 222, respectively) that we used for nested PCR. Genomic sequences determined in this study have been deposited in GenBank. Resulting amplicon sequences were compared by pairwise VP1 nucleotide sequence with homologous sequences of the VP1 retrieved from the GenBank database using the program BLAST in NCBI (October 2016). Each sample was assigned the type with the highest identity score. Types were confirmed with the RIVM genotyping tool (http://www.rivm.nl/mpf/enterovirus/typingtool/). All sequences were aligned by using ClustalW multiple alignment program within the BioEdit Sequence Alignment Editor package, version 7.0.9.0. Phylogenetic studies and genetic distances were calculated by using the Molecular Evolutionary Genetics Analysis (MEGA5) software package (http://megasoftware.net/) as described in the legends to the figures. Trees were inferred with a Neighbor-Joining method. Distances were computed using the Kimura 2-parameter model. The robustness of the nodes was tested by 1000 bootstrap replications. Bootstrap support values >80 are shown in nodes. Genetic distances were determined using the p-distance model for nucleotide and deduced amino acid sequences.
